# Microsaccades: Empirical Research and Methodological Advances - Introduction to Part 1 of the Thematic Special Issue

**DOI:** 10.16910/jemr.12.6.1

**Published:** 2020-06-19

**Authors:** Susana Martinez-Conde, Ralf Engbert, Rudolf Groner

**Affiliations:** State University of New York (SUNY) Downstate Health Sciences University, USA; University of Potsdam, Germany; University of Bern and SciAns GmbH, Iffwil, Switzerland

**Keywords:** microsaccades, sensory processes, perceptual processes, cognitive processes, high-speed eye tracking, fixational eye movements

## Abstract

Recent technical developments and increased affordability of high-speed eye tracking devices have brought microsaccades to the forefront of research in many areas of sensory, perceptual, and cognitive processes. The present thematic issue on “Microsaccades: Empirical Research and Methodological Advances” invited authors to submit original research and reviews encompassing measurements and data analyses in fundamental, translational, and applied studies.

We present the first volume of this special issue, comprising 14 articles by research teams around the world. Contributions include the characterization of fixational eye movements and saccadic intrusions in neurological impairments and in visual disease, methodological developments in microsaccade detection, the measurement of fixational eye movements in applied and ecological scenarios, and advances in the current understanding of the relationship between microsaccades and cognition.

When fundamental research on microsaccades experienced a renaissance at the turn of the millennium (c.f. 4), one could hardly have been so bold as to predict the manifold applications of research on fixational eye movements in clinic and practice. Through this great variety of areas of focus, some main topics emerge.

One such theme is the applicability of microsaccade measures to neurological and visual disease. Whereas microsaccade quantifications have been largely limited to participants with intact visual and oculomotor systems, recent research has extended this interest into the realm of neural and ophthalmic impairment (see 1 for a review). In this volume, Becker et al analyze “ Saccadic intrusions in amyotrophic lateral sclerosis (ALS)” and Kang et al study “ Fixational eye movement waveforms in amblyopia”, delving into the characteristics of fast and slow eye movements. Two other articles focus on how the degradation of visual information, which is relevant to many ophthalmic pathologies, affects microsaccadic features. Tang et al investigate the “ Effects of visual blur on microsaccades on visual exploration” and conclude that the precision of an image on the fovea plays an important role in the calibration of microsaccade amplitudes during visual scanning. Otero-Millan et al use different kinds of visual stimuli and viewing tasks in the presence or absence of simulated scotomas, to determine the contributions of foveal and peripheral visual information to microsaccade production. They conclude that “ Microsaccade generation requires a foveal anchor”.

The link between microsaccadic characteristics and cognitive processes has been a mainstay of microsaccade research for almost two decades, since studies in the early 2000s connected microsaccade directions to the spatial location of covert attentional cues (2 3). In the present volume, Dalmaso et al report that “ Anticipation of cognitive conflict is reflected in microsaccades”, providing new insights about the top-down modulation of microsaccade dynamics. Ryan et al further examine the relationship between “ Microsaccades and covert attention” during the performance of a continuous, divided-attention task, and find preliminary evidence that microsaccades track the ongoing allocation of spatial attention. Krueger et al discover that microsaccade rates modulate with visual attention demands and report that “ Microsaccades distinguish looking from seeing”. Taking the ecological validity of microsaccade investigations one step further, Barnhart et al evaluate microsaccades during the observation of magic tricks and conclude that “ Microsaccades reflect the dynamics of misdirected attention in magic”.

Two articles examine the role of individual differences and intraindividual variability over time on microsaccadic features. In “ Reliability and correlates of intra-individual variability in the oculomotor system” Perquin and Bompas find evidence for intra-individual reliability over different time points, while cautioning that its use to classify self-reported individual differences remains unclear. Stafford et al provide a counterpoint in “ Can microsaccade rate predict drug response?” by supporting the use of microsaccade occurrence as both a trait measure of individual differences and as a state measure of response to caffeine administration.

Methodological and technical advances are the subjects of three papers in this volume. In “ Motion tracking of iris features to detect small eye movements” Chaudhary and Pelz describe a new video-based eye tracking methodology that relies on higher-order iris texture features, rather than on lower-order pupil center and corneal reflection features, to detect microsaccades with high confidence. Munz et al present an open source visual analytics system called “ VisME: Visual microsaccades explorer” that allows users to interactively vary microsaccade filter parameters and evaluate the resulting effects on microsaccade behavior, with the goal of promoting reproducibility in data analyses. In “ What makes a microsaccade? A review of 70 years research prompts a new detection method” Hauperich et al review the microsaccade properties reported between the 1940s and today, and use the stated range of parameters to develop a novel method of microsaccade detection.

Lastly, Alexander et al switch the focus from the past of microsaccade research to its future, by discussing the recent and upcoming applications of fixational eye movements to ecologically-valid and real-world scenarios. Their review “ Microsaccades in applied environments: real-world applications of fixational eye movement measurements” covers the possibilities and challenges of taking microsaccade measurements out of the lab and into the field.

Microsaccades have engaged the interest of scientists from different backgrounds and disciplines for many decades and will certainly continue to do so. One reason for this fascination might be microsaccades’ role as a link between basic sensory processes and high-level cognitive phenomena, making them an attractive focus of interdisciplinary research and transdisciplinary applications. Thus, research on microsaccades will not only endure, but keep evolving as the present knowledge base expands. Part 2 of the special issue on microsaccades is already in progress with articles currently under review and will be published in 2021.
